# Effects of radioiodine administration on serum concentrations of matrix metalloproteinases, adiponectin and thrombospondin-1

**DOI:** 10.1186/1756-6614-6-9

**Published:** 2013-08-06

**Authors:** Andrzej Lewinski, Anna Brona, Krzysztof C Lewandowski, Diana Jedrzejuk, Anna Bohdanowicz-Pawlak, Elżbieta Skowronska-Jozwiak, Małgorzata Bienkiewicz, Andrzej Milewicz

**Affiliations:** 1 Department of Endocrinology & Metabolic Diseases, Medical University of Lodz, Polish Mother’s Memorial Hospital – Research Institute, Rzgowska 281/289, 93-338 Lodz, Poland; 2 Department of Endocrinology, Diabetes & Isotope Therapy, Medical University of Wroclaw, Wroclaw, Poland; 3 Department of Quality Control & Radiation Protection, Medical University of Lodz, Lodz, Poland

**Keywords:** Metalloproteinases, Adiponectin, Thrombospondin, Thyrotoxicosis, Radioactive iodine

## Abstract

**Background:**

In order to assess safety of radioactive iodine administration in the treatment of thyrotoxicosis, we measured concentrations of matrix metalloproteinase-2 (MMP-2), its main inhibitor – TIMP-2 (tissue inhibitor of MMP-2), matrix metalloproteinase-9 (MMP-9), its main inhibitor – TIMP-1, adiponectin, as well as pro-inflammatory and procancerogenic thrombospondin-1 (TSP-1).

**Design and patients:**

The study involved 23 patients treated with radioiodine for thyrotoxicosis. Serum concentrations of TSH, free T4, free T3, MMP-2, MMP-9, TIMP-1, TIMP-2, total adiponectin and TSP-1 were measured by immunoassays just before radioiodine administration (visit 1), and subsequently, after 7 days (visit 2), 3 months (visit 3), 6 to 8 months (visit 4) and 15–18 months after radioiodine administration (visit 5).

**Results:**

There were no acute changes in serum concentrations of MMP-2, MMP-9, TIMP-1, TIMP-2, adiponectin and TSP-1 (visit 1 *vs.* 2). Subsequently, there was an increase in MMP-2 (from 393±106 ng/ml to 774±424 ng/ml), TIMP-1 (from 177±76 ng/ml to 296±118 ng/ml), and adiponectin (from 16442±9490 ng/ml to 23518±9840 ng/ml), visit 1 to 5, respectively (p < 0.01). Further analysis revealed no significant change in MMP-2/TIMP-2 ratio, but there was a significant decrease in MMP-9/TIMP-1 ratio (p < 0.05), suggestive of possible decrease in free MMP-9 concentrations.

**Conclusions:**

Our data reveal a significant and sustained increase in serum adiponectin, as well as possible decrease of free MMP-9 concentration after radioiodine administration. In contrast, there was no significant change of TSP-1. This might indicate overall safety of radioiodine treatment of thyrotoxicosis in terms of the risks of subsequent cardiovascular and neoplastic disease.

## Background

The term matrix metalloproteinases (MMPs) refers to a group of enzymes that physiologically remodel extracellular matrix, but also contribute to development of various pathological states, such as neoplasms, inflammatory and cardiovascular diseases [1]. Furthermore, increased activity of MMPs in blood vessels has been implicated in formation of aneurysms [2], while formation of unstable atherosclerotic plaques, as a result of locally increased activity of MMPs, might in turn lead to an increased risk thrombotic and embolic events, including myocardial infarctions and strokes [3-5].

Adiponectin is a plasma protein secreted from adipocytes in relatively large amounts, where decreased adiponectin secretion has been directly linked with the development of type 2 diabetes mellitus and metabolic syndrome, known to be associated with an increased risk of cardiovascular disease. There is ample evidence demonstrating that adiponectin has anti-inflammatory, anti-atherosclerotic and vasoprotective actions, affects signaling in myocardial cells and exerts beneficial actions on the heart after pressure overload and ischemia-reperfusion injury [6,7].

Thrombospondin-1 (TSP-1) is a member of a family of five (5) structurally related extracellular glycoproteins that plays a major role in cell-matrix and cell-cell interactions. TSP-1 is highly expressed in obese, insulin-resistant subjects. When released to the matrix, matricellular proteins associate with growth factors, cytokines, and other bioactive effectors and bind to cell surface receptors transducing signaling cascades. TSP-1 is highly correlated with adipose inflammation [8]; and is decreased by pioglitazone [9], though there is also evidence of possible protective properties in circumstances, such as cardiac remodeling after injury [10]. Thrombospondin has been also found to be secreted by thyrocytes in a pattern that is opposite to thyroglobulin [11], and subsequently TSP-1 has also been identified as a potential regulator of angiogenesis and tumour progression [12].

Recent studies [13,14], demonstrated that even subclinical thyrotoxicosis may be independently associated, with an increased cardiovascular morbidity and mortality, though there are still several issues pertaining this subject [15]. Pharmacological treatment of thyrotoxicosis occasionally causes agranulocytosis, and this complication, unfortunately, cannot be prevented by routine full blood count monitoring of asymptomatic patients [16]. Pharmacological treatment of thyrotoxicosis has other drawbacks, as it generally does not lead to permanent cure in subjects with toxic multinodular goitre, while relapse in subjects with Graves’ disease may exceed 50 per cent. For these reasons radioactive iodine treatment (RIT) has been used to treat thyrotoxicosis since 1940's. In the USA, and less commonly in Europe, RIT is prescribed even for children with Graves’s disease [17]. There are, however, some recent data [18], suggestive of an increase in cardiovascular and cancer mortality after RIT, at least in some subjects, though a definite causality remains to be proven [19]. Given potential option of surgery, the safety of RIT is of paramount importance from the clinical view-point. For reasons outlined above, we have endeavoured to prospectively investigate whether serum concentrations of selected risk markers of cardiovascular and neoplastic diseases, such as matrix metalloproteinases (MMP-2, MMP-9), their inhibitors (TIMP-1, TIMP-2), adiponectin and TSP-1 might change following radioiodine treatment of thy-rotoxicosis.

## Design and patients

The study involved 23 patients (three males) age 53±12 (mean±SD), BMI 26.5±4.6 kg/m^2^ years treated with radioiodine for thyrotoxicosis. Serum concentrations TSH, free T4, free T3, MMP-2, MMP-9, TIMP-1, TIMP-2, total adiponectin and TSP-1 were measured just before radioiodine administration (visit 1), and subsequently, after 7 days (visit 2), 3 months (visit 3), 6 to 8 months (visit 4) and 15–18 months after radioiodine administration (visit 5).

Radioactive iodine was administered according to the protocol that involved thyroid goitre or nodule volume and radioiodine uptake (T24) and radioiodine activity for 1.0 gram of thyroid tissue in thyroid with dose depending on the type of thyroid disease. In this method the formula applied for calculation of the dose of radioiodine was as follows [20]:

$$\begin{array}{l} \mathrm{Dose}\;\mathrm{of}\;\mathrm{radioiodine}\;\left(\mathrm{mCi}\right)\\ \;=\frac{\mathrm{Thyroid}\;\mathrm{mass}\;\left(g\right)\times \mathrm{radioiodine}\;\mathrm{activity}\;\mathrm{for}\;1.0\;g\;\mathrm{of}\;\mathrm{thyroid}\;\mathrm{tissue}\;\left(\mathrm{\mu Ci}/g\right)}{T24\;\left(\%\right)\times 10} \end{array}$$

where:

-1.0 g of thyroid tissue mass means 1.0 ml of thyroid volume,

radioiodine activity to be administered in adults: in Graves’ disease 80–150 μCi/1.0 g of thyroid tissue, in toxic thyroid nodule 150 μCi/1.0 g of thyroid tissue, in toxic goitre 100-150 μCi/1.0 g of thyroid tissue [21],

T24 (%) - radioiodine uptake [20].

Measurements of MMP-2, MMP-9, TIMP-1, TIMP-2, adiponectin and TSP-1 were performed by R & D systems immunoassays (Human Quantikine ELISA kit, for MMPs and TIMPs, Human Total Adiponectin /Acrp30 Quantikine ELISA Kit, human TSP-1 Quantikine ELISA Kit - catalogue numbers: DMP2F0 for MMP-2, DMP900 for MMP-9, DTM100 for TIMP-1, DTM200 for TIMP-2, DRP300 for adiponectin and DTSP10 for TSP-1).

### Statistical analysis

Given the dependent character of the data (repeated measurements in the same subjects), the data were analysed by means of simple descriptive statistics of location and dispersion as well as Friedman ANOVA for dependent samples. If the observed difference between all measurements was significant *post hoc* Tukey’s test was performed. Statistical significance was considered to be achieved for p ≤ 0.05. All the calculations were performed by means of Statistica v 9.0 computer software.

The study was approved by the Ethics Committee of the Medical University of Lodz, Poland.

## Results

**Figure 1:**
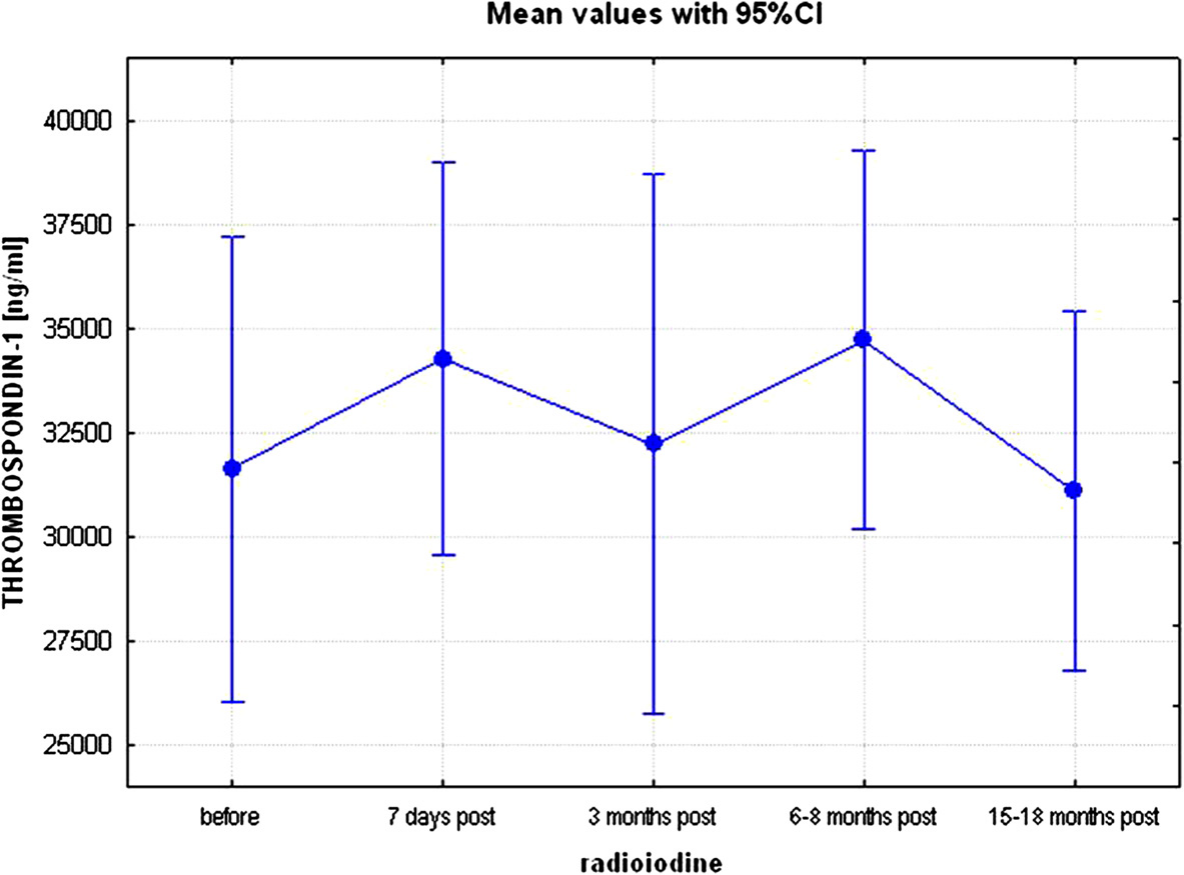
Concentrations of thrombospondin-1 (TSP-1) before and after radioiodine administration at consecutive time-points (visits 1–5), p=0.72 (Kruskal-Wallis’ ANOVA for repeated measures design).

**Figure 2:**
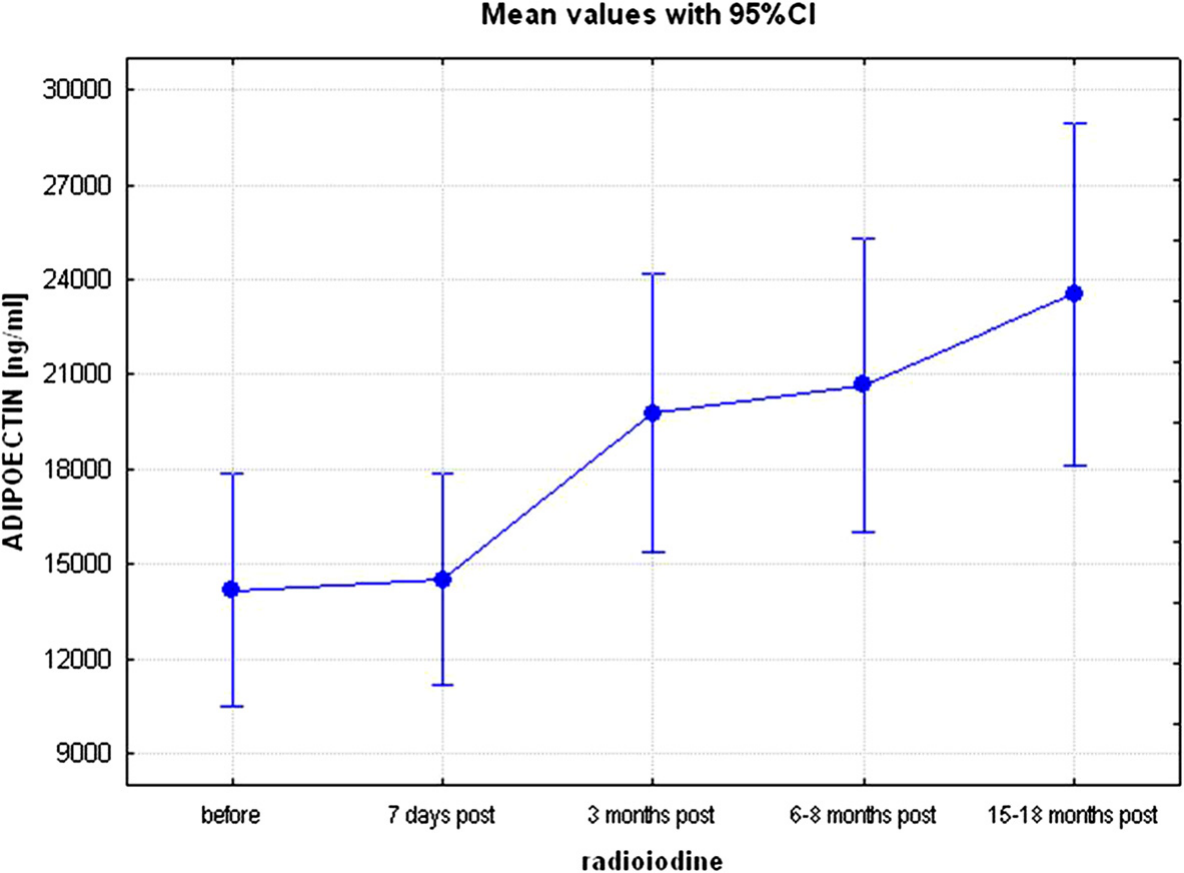
Concentrations of adiponectin before and after radioiodine administration at consecutive time-points (visits 1–5), p < 0.01 (Kruskal-Wallis’ ANOVA for repeated measures design).

**Figure 3:**
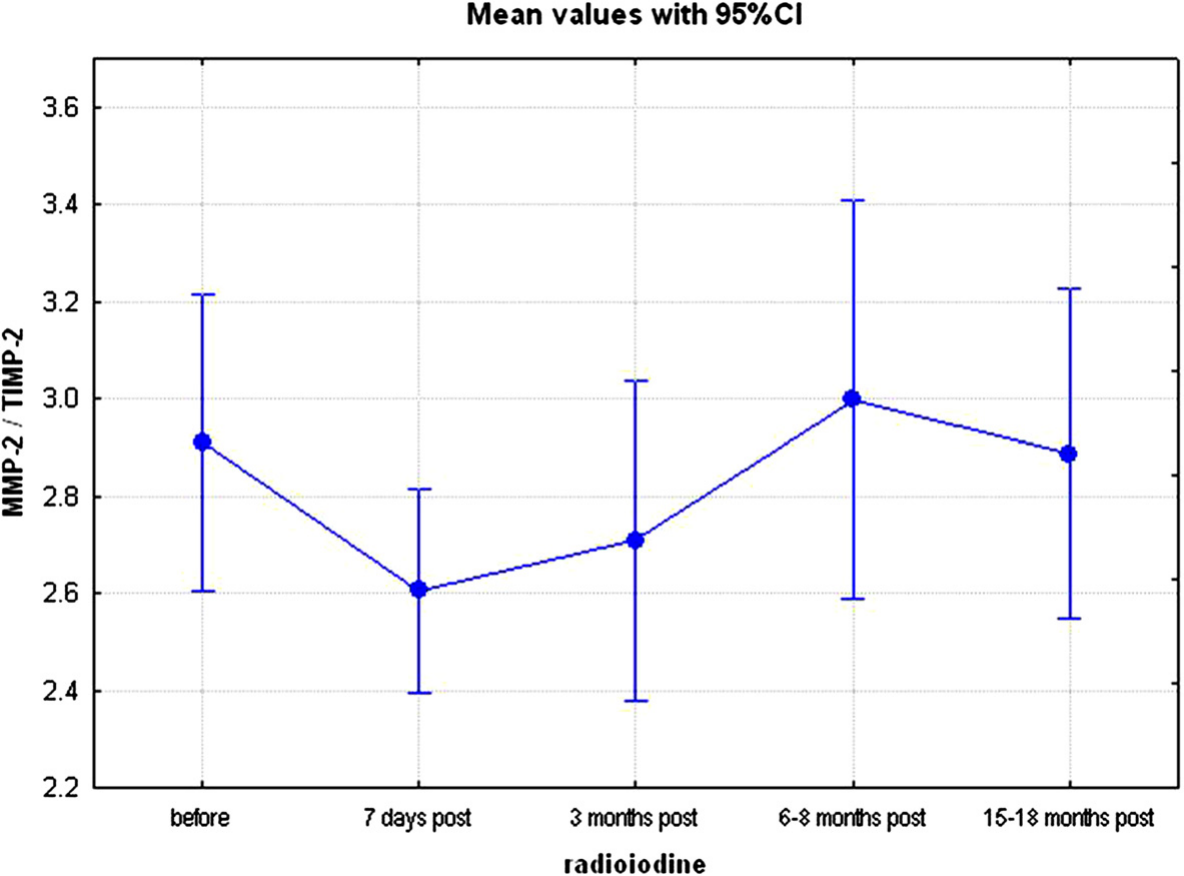
**The ratio of concentrations of matrix metalloproteinases and their respective inhibitors at consecutive time-points (visits 1–5).** Matrix metalloproteinase-2 (MMP-2) to tissue inhibitor of matrix metalloproteinase-2 (TIMP-2) ratio, p = 0.34 (Kruskal-Wallis’ ANOVA for repeated measures design) before radioiodine administration and at all subsequent time-points.

**Figure 4:**
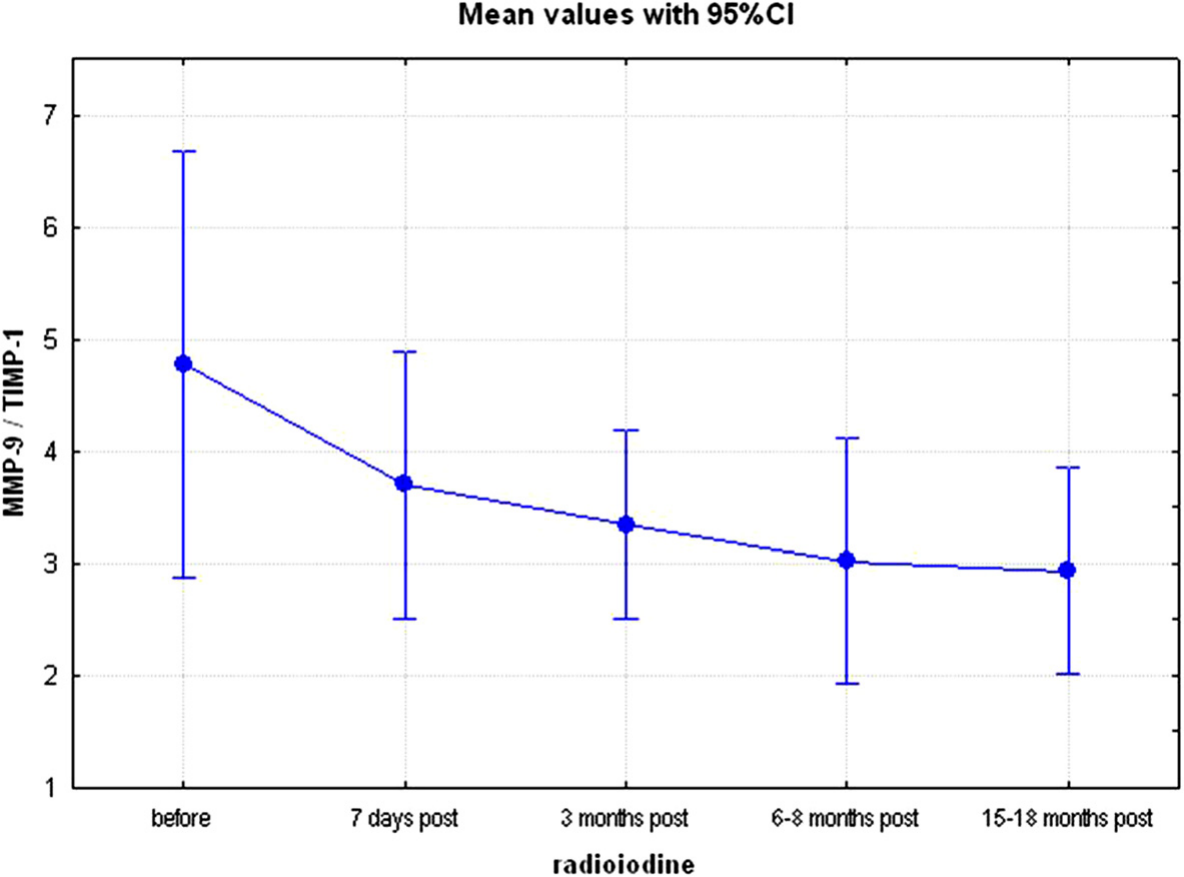
**The ratio of concentrations of matrix metalloproteinases and their respective inhibitors at consecutive time-points (visits 1–5).** Matrix metalloproteinase-9 (MMP-2) to tissue inhibitor of matrix metalloproteinase-1 (TIMP-1) ratio, p < 0.05 (Kruskal-Wallis’ ANOVA for repeated measures design) before radioiodine administration and at all subsequent time-points.

Results of the study are presented in Tables 1, 2 and 3 and Figures 1, 2, 3 and 4. Following radioiodine treatment there was a fall in free T4 between visit 2 and visit 3 (p < 0.01), however, as patients later developing hypothyroidism were treated with L-thyroxine, then the concentrations of free T4 remained stable at subsequent visits. Changes of TSH, free T4, free T3, glucose and lipids at the beginning and at the end of the study are presented in Table 1. Concentrations of other parameters, measured at subsequent visits (1–5) are presented in Table 2. There were no acute changes in serum concentrations of MMP-2, MMP-9, TIMP-1, TIMP-2, adiponectin and TSP-1 (visit 1 *vs.* visit 2). There was no significant change in serum concentrations of TSP-1 throughout the study (Table 2, Figure 1). In contrast to TSP-1, there was, however, an increase in serum adiponectin (already significant at visit 3, p < 0.05), that remained significant for further duration of the study and (16442±9490 ng/ml at visit 1 (before radioiodine administration) *vs.* to 23518±9840 ng/ml, at visit 5 (15–18 months after radioiodine administration), p < 0.01, Table 2, Figure 2.

**Table 1:** **Concentrations of TSH, free T4, free T3, glucose and lipids at the beginning and at the end of the study (visit 1 *vs. *visit 5)**

	**Before radioiodine (Visit 1)**		**15-18 months post radioiodine (Visit 5)**		***p- value***
	**Mean**	**SD**	**Mean**	**SD**	
TSH* [μIU/ml]	0.10	0.20	3.74	3.15	***0.0003***
Free T4* [pmol/l]	26.8	15.6	14.42	2.23	***0.0008***
Free T3* [pmol/l]	10.1	5.3	4.87	1.01	***0.0003***
Glucose [mg/dl]	93	13	90	12	*0.62*
Total cholesterol [mg/dl]	197	37	237	56	***0.012***
LDL-cholesterol [mg/dl]	108	38	153	54	***0.001***
HDL-cholesterol [mg/dl]	61	17	61	14	*0.16*
Triglycerides [mg/dl]	121	118	116	58	*0.21*

^*^Reference ranges: free T4 11.5-22.7 pmol/l, free T3 2.76-6.45 pmol/l, TSH 0.4-4 μIU/ml 1 pmol/l x 0.0777= ng/dl.

**Table 2:** Descriptive statistics for serum concentrations of matrix metaloproteinases (MMP-2, MMP-9), tissue inhibitors of matrix metalloproteinases (TIMP-1, TIMP-2), adiponectin and thrombospondin-1 (TSP-1) at five (5) consecutive visits – before and after radioiodine therapy, presented with probability values (p-values) of Kruskal-Wallis’ ANOVA for repeated measures design

	**VISIT:**	**n**	**Mean ± SD**	***p-value***
MMP-2 [ng/ml]	before radioiodine	23	393 ± 106	***0.001***
	7 days post	23	373 ± 88	
	3 months post	23	423 ± 115	
	6 - 8 months post	23	452 ± 124	
	15-18 months post	17	774 ± 424	
MMP-9 [ng/ml]	before radioiodine	23	631 ± 327	*0.68*
	7 days post	23	594 ± 353	
	3 months post	23	704 ± 364	
	6 - 8 months post	23	714 ± 430	
	15-18 months post	17	470 ± 106	
TIMP-1 [ng/ml]	before radioiodine	23	177 ± 76	***0.00001***
	7 days post	23	186 ± 54	
	3 months post	23	215 ± 86	
	6 - 8 months post	23	263 ± 128	
	15-18 months post	17	296 ± 118	
TIMP-2 [ng/ml]	before radioiodine	23	136 ± 44	*0.36*
	7 days post	23	143 ± 36	
	3 months post	23	161 ± 39	
	6 -8 months post	23	153 ± 39	
	15-18 months post	17	168 ± 41	
Adiponectin [ng/ml]	before radioiodine	23	16442 ±9490	***0.001***
	7 days post	23	14430 ± 6216	
	3 months post	23	21300 ± 8908	
	6 - 8 months post	23	20005 ± 8056	
	15-18 months post	17	23518 ± 9840	
TSP-1 [ng/ml]	before radioiodine	23	31246 ± 10680	*0.72*
	7 days post	23	32761 ± 9046	
	3 months post	23	32507 ± 10892	
	6 - 8 months post	23	33751 ± 8830	
	15-18 months post	17	31758 ± 8319	

**Table 3:** Descriptive statistics for MMP-2/TIMP-2 ratio and MMP-9/TIMP-1 ratio at five (5) consecutive visits – before and after radioiodine therapy, presented with probability values (p-values) of Kruskal-Wallis’ ANOVA for repeated measures design

	**VISIT:**	**n**	**Mean ± SD**	***p-value***
MMP-2/TIMP-2	before radioiodine	23	2.96 ± 0.54	*0.34*
	7 days post	21	2.65 ± 0.43	
	3 months post	23	2.68 ± 0.57	
	6 - 8 months post	23	3.00 ± 0.65	
	15-18 months post	17	2.86 ± 0.63	
MMP-9/TIMP-1	before radioiodine	23	4.16 ± 3.19	***0.042***
	7 days post	21	3.40 ± 2.06	
	3 months post	23	3.39 ± 1.47	
	6 - 8 months post	23	3.00 ± 1.95	
	15-18 months post	17	2.92 ± 1.68	

Analysis of concentrations of MMPs and their inhibitors revealed no significant change in serum MMP-9 throughout the study, but an increase in MMP-2 (from 393±106 ng/ml, to 774±424 ng/ml) and TIMP-1 (from 177±76 ng/ml to 296±118 ng/ml), visit 1 to visit 5 respectively, p < 0.01. Further analysis, presented in Table 3, Figures 3 and 4, revealed, however, no significant change in MMP-2/TIMP-2 ratio. In contrast, there was a significant decrease in MMP-9/TIMP-1 ratio (p < 0.05), suggestive of possible decrease in concentrations of free MMP-9.

## Discussion

To the best of our knowledge, this is the first study where concentrations of TSP-1 were assessed before and after treatment with radioiodine. Though this is a negative observation, the lack of any significant change of serum TSP-1 after treatment with radioiodine is indeed highly reassuring. TSP-1 which represents, besides thyroglobulin, the main protein secreted by thyroid cells has been found to play a role in the process of folliculogenesis [22], and inhibition of apoptosis of thyroid cells [23]. Furthermore, it has recently been found that TSP-1 directly promotes progression of papillary thyroid carcinoma, and namely *BRAF(V600E)*, i.e. the most common somatic mutation in papillary thyroid carcinoma, requires activation of several other genes, including TSP-1, in order to facilitate tumor invasion and metastasis [24,25]. Sid et al. [26] also demonstrated that the aggressive behavior of human thyroid malignant cells was closely correlated to the amount of TSP-1. They also demonstrated that exogenously added TSP-1 stimulated by two-fold the invasiveness of follicular thyroid carcinoma cells, while the use of specific anti-TSP-1 blocking antibodies led to a drastic inhibition of the basal follicular thyroid carcinoma cell invasion. In such context, the lack of any significant change in serum TSP-1 might signify that radioiodine treatment of thyrotoxicosis is unlikely to influence the development of subsequent thyroid cancer. It should be, however, strongly emphasized that that the observed lack of any significant changes in serum concentrations of TSP-1, must be also confirmed on a tissue levels, i.e. by studies of expression of TSP-1 following radioiodine treatment in thyroid cells.

Our study is also an extension of our previous report [27], where we demonstrated an unequivocal rise in serum adiponectin at three months after radioiodine administration. In this paper we have prospectively carried out our observations up to 15–18 months after radioiodine treatment, and so we have demonstrated that raised adiponectin concentrations persisted at all subsequent time-points. In our opinion, this implies that an increase in serum adiponectin is a sustained phenomenon, while longitudinal and prospective nature of our study diminishes the chance of possible type I (i.e., false positive) statistical error. The observed sustained increase in vasoprotective adiponectin is reassuring, given some recent controversies regarding cardiovascular safety of treatment with radioactive iodine. As mentioned above [6,7], adiponectin improves insulin sensitivity and exerts anti-atherosclerotic effects in blood vessels. Indeed there are some studies [28] suggestive that the plasma leptin/adiponectin ratio predicts first cardiovas-cular event at least in men. Furthermore many cancer cell lines express adiponectin receptors, and adiponectin *in vitro* limits cell proliferation and induces apoptosis. Recent *in vitro* studies demonstrate the antiangiogenic and tumor growth-limiting properties of adiponectin [29]. It is to be recalled, however, that the significance of total adiponectin concentrations as a marker of a risk of cardiovascular disease has been recently questioned [30,31].

Current study has also helped to clarify issues associated with changes of concentrations of MMPs and their inhibitors following radioiodine administration. In particular, we have demonstrated that though there was a significant increase in concentrations of MMP-2, that was no significant change in MMP-2/TIMP-2 ratio; the latter possibly being a result of a concomitant (though not significant) increase in TIMP-2 concentrations (from 136±44 ng/ml to 168±41 ng/ml). In contrast, there was no change in serum MMP-9 concentrations but there was a significant increase in TIMP-1 concentrations, with subsequent fall in MMP-9/TIMP-1 ratio. It should be mentioned that TIMP-2 is the principal inhibitor of MMP-2, while TIMP-1 is the principal inhibitor of MMP-9 [32], hence these results are suggestive of possible fall in free (i.e., biologically active) MMP-9 concentrations following radioiodine treatment. Interestingly, the observed increase of serum TIMP-1 concentrations seems to be independent of TSP-1, even though there is some evidence that TSP-1 induced expression of TIMP-1 in follicular thyroid carcinoma cells [33]. Therefore, our data support a notion - expressed previously - that treatment with radioactive iodine appears safe [34], as evidenced in children treated with radioactive iodine for Graves’ disease [35]. We have, already mentioned in our previous paper [27] that cardiovascular safety of radioactive iodine would also depend on meticulous follow-up of patients who undergo this treatment, as efforts must be made to detect and to treat radioiodine-induced hypothyroidism. If this is not done properly, then hypothyroidism *per se* might increase a risk of subsequent cardiovascular disease [36-38]. It should also be mentioned that stable concentrations of MMPs, with possible fall of free MMP-9, do not support a notions of significant involvement of MMPs in the development of neoplasms following treatment with radioactive iodine. In this context, we note that large study of Ron et al. [39], based on a data from 35 593 subjects, failed to reveal an increase in cancer mortality in subjects treated with radioiodine.

## Conclusions

In summary, results of our study are reassuring in terms cardiovascular and neoplastic safety of radioiodine treatment of thyrotoxicosis. In particular, administration of radioiodine does not alter serum concentrations of proinflammatory and procancerogenic TSP-1, and is associated with a sustained increase in serum adiponectin. Furthermore, there is no change in MMP-2/TIMP-2 ratio, with an increase in TIMP-1, and a fall of MMP-9/TIMP-1 ratio is recorded, the latter possibly indicative of a fall in free MMP-9 concentrations. However, it must be stressed that in our study we assessed concentrations of indirect markers associated with the risk of cardiovascular and neoplastic diseases, which cannot be regarded as surrogates of hard clinical end-points, such as morbidity and mortality.

## Competing interests

The authors declare that they have no competing interests.

## Authors’ contributions

AL designed and coordinated the study, and revised the text of manuscript, AB supervised the radioiodine therapy of patients in acquisition of data, KCL participated in coordination of the study and drafted the manuscript, DJ, AB-P and ES-J participated in acquisition of data, MB performed statistical analysis of results, AM conceived the study and participated in design of manuscript. All authors have read and approved the final manuscript.
